# Manipulation of HSP70-SOD1 Expression Modulates SH-SY5Y Differentiation and Susceptibility to Oxidative Stress-Dependent Cell Damage: Involvement in Oxotremorine-M-Mediated Neuroprotective Effects

**DOI:** 10.3390/antiox12030687

**Published:** 2023-03-10

**Authors:** Miriana Scordino, Monica Frinchi, Giulia Urone, Domenico Nuzzo, Giuseppa Mudò, Valentina Di Liberto

**Affiliations:** 1 Dipartimento di Biomedicina, Neuroscienze e Diagnostica Avanzata, Università di Palermo, Corso Tukory 129, 90134 Palermo, Italy; 2 Istituto per la Ricerca e l’Innovazione Biomedica (IRIB), Consiglio Nazionale delle Ricerche (CNR), via U. La Malfa 153, 90146 Palermo, Italy

**Keywords:** oxotremorine, muscarinic acetylcholine receptor, KNK437, neuroprotection, heat shock proteins, superoxide dismutase

## Abstract

The differentiation of neural progenitors is a complex process that integrates different signals to drive transcriptional changes, which mediate metabolic, electrophysiological, and morphological cellular specializations. Understanding these adjustments is essential within the framework of stem cell and cancer research and therapy. Human neuroblastoma SH-SY5Y cells, widely used in neurobiology research, can be differentiated into neuronal-like cells through serum deprivation and retinoic acid (RA) supplementation. In our study, we observed that the differentiation process triggers the expression of Heat Shock Protein 70 (HSP70). Notably, inhibition of HSP70 expression by KNK437 causes a dramatic increase in cell death. While undifferentiated SH-SY5Y cells show a dose-dependent decrease in cell survival following exposure to hydrogen peroxide (H_2_O_2_), differentiated cells become resistant to H_2_O_2_-induced cell death. Interestingly, the differentiation process enhances the expression of SOD1 protein, and inhibition of HSP70 expression counteracts this effect and increases the susceptibility of differentiated cells to H_2_O_2_-induced cell death, suggesting that the cascade HSP70-SOD1 is involved in promoting survival against oxidative stress-dependent damage. Treatment of differentiated SH-SY5Y cells with Oxotremorine-M (Oxo), a muscarinic acetylcholine receptor agonist, enhances the expression of HSP70 and SOD1 and counteracts tert–Butyl hydroperoxide-induced cell death and reactive oxygen species (ROS) generation. It is worth noting that co-treatment with KNK437 reduces SOD1 expression and Oxo-induced protection against oxidative stress damage, suggesting the involvement of HSP70/SOD1 signaling in this beneficial effect. In conclusion, our findings demonstrate that manipulation of the HSP70 signal modulates SH-SY5Y differentiation and susceptibility to oxidative stress-dependent cell death and unravels novel mechanisms involved in Oxo neuroprotective functions. Altogether these data provide novel insights into the mechanisms underlying neuronal differentiation and preservation under stress conditions.

## 1. Introduction

Neuronal differentiation is a complex process modulated by the integrated action of several mechanisms, which drive cellular morphological and functional changes. Among them, the spatial and temporal fine adjustment of transcription and protein synthesis plays a major role [1]. A more in-depth comprehension of the molecular changes underlying this process can provide important clues for the development of innovative strategies to enhance neuronal differentiation and maturation from stem cells. This information can be crucial in the context of neurodegenerative disease therapy.

Human SH-SY5Y neuroblast-like cells are broadly used for in vitro investigation in the field of neurobiology research to overcome all the limitations associated with the culture of primary neurons from the embryonic brain [2,3]. SH-SY5Y cells can be differentiated from a neuroblast-like state (undifferentiated cells) into a neuron-like phenotype (differentiated cells) by using different protocols, including supplementation of retinoic acid (RA) and specific neurotrophins, such as brain-derived neurotrophic factor (BDNF) [4]. Interestingly, the use of precise protocols for the differentiation of SH-SY5Y cells can select specific neuronal subtypes, including dopaminergic, cholinergic, and adrenergic neurons [5].

Although SH-SY5Y cells, both undifferentiated and differentiated, are used almost indiscriminately for in vitro experiments that require neuron-like cells, several investigations have described significant differences between the two phenotypes [4]. Undifferentiated SH-SY5Y cells display a non-polarized morphology with very few short processes. They usually grow fast in aggregates and express markers of immature neurons [6,7]. When differentiated, SH-SY5Y cells decrease the proliferation rate, elongate branched neurite projections, and express markers of mature neurons, such as neuronal nuclear protein (NeuN) and microtubule-associated protein (MAP) [5,8,9,10].

Interestingly, these morphological differences are coupled to specific functional specializations, including the generation of action potentials [11] and the resistance to hyperthermic stress that depends on the increased expression of Heat Shock Proteins 70 (HSP70) during the differentiation process [12].

HSPs are molecular chaperones involved in maintaining the structure of proteins under different stress conditions, including disruption of osmotic homeostasis, nutrient deprivation, UV exposure, pathogen infection, and oxidative stress [13,14,15]. Within the framework of redox homeostasis, HSP70 works as an ATP-dependent enzyme, which protects enzymes and other proteins from the effects of reactive species [16]. Moreover, HSP70 signaling induces the expression of antioxidant enzymes, including superoxide dismutase (SOD) [17,18]. Three isoforms of SOD have been identified in mammalian cells: SOD1, mainly located in the cytosol; SOD2, prevalently localized in the mitochondria; and the extracellular SOD3 [19].

HSP70 expression can be induced by different stimuli, including stress and exposure to natural and synthetic molecules [20]. In this context, we have recently demonstrated that Oxotremorine-M (Oxo), a muscarinic acetylcholine receptor (mAchR) agonist, increases HSP70 protein levels in the rat hippocampus [21].

In this study, we investigated whether the expression of HSP70 may play a role in the SH-SY5Y cell differentiation process and in cell response to oxidative stress-dependent damage, with a focus on the involvement of SOD1. In addition, we explored whether Oxo treatment may protect SH-SY5Y cells from oxidative stress-induced cell death by modulating HSP70 and SOD1 expression.

## 2. Materials and Methods

### 2.1. SH-SY5Y Culture and Differentiation

Neuroblastoma SH-SY5Y cells were cultured in T25 tissue culture flasks in complete Dulbecco’s Modified Eagle’s Medium and F12 (Corning, DMEM/F12; 1:1) supplemented with 10% fetal bovine serum (FBS), 100 U/mL penicillin and 100 U/mL streptomycin, and 2 mM l-glutamine% (all purchased from Gibco, ThermoFisher Scientific). Cells were maintained in a humidified atmosphere of 95% air and 5% CO_2_ at 37 °C, as described in [22]. Cell growth medium was changed every 3 days, and the cells were sub-cultured once they reached 85–90% confluence.

The differentiation protocol was initiated the day after plating by replacing growth medium with differentiation medium containing Neurobasal, 2% B27 Supplement, 100 U/mL penicillin and 100 U/mL streptomycin, Glutamax 1% (all purchased from Gibco, ThermoFisher Scientific, Waltham, MA USA) plus 10 μM trans-retinoic acid (tRA, R2625 Merck KGaA, Darmstadt, Germany). Cell differentiation medium was changed every 3 days, and the development of neuron-like morphology was monitored every day for up to 11 days.

### 2.2. Cell Treatments

In order to assess the impact of inhibition of HSP70 expression by KNK437 (SC-221709 Santa Cruz Biotechnology, 50 µM) on viability of fully differentiated SH-SY5Y cells, KNK437 was added to the cell medium at the differentiation day 4, 8, or 10, and MTT test was performed at day 11.

Sensitivity of undifferentiated or fully differentiated SH-SY5Y cells to H_2_O_2_-induced cell death was assessed in cells exposed for 24 h to different H_2_O_2_ doses, ranging between 50 µM and 1 mM. MTT test was performed in undifferentiated cells 4 days after plating and in SH-SY5Y cells differentiated for 11 days.

The impact of HSP70 expression on fully differentiated SH-SY5Y cell protection against H_2_O_2_-induced cell death was explored by treating cells first with KNK437 (50 µM) at day 8 of differentiation and then with H_2_O_2_ (200 µM) at day 10 (without removing KNK437 from the culture medium). MTT test was performed 24 h later, on day 11 of differentiation.

Expression of HSP70 in fully differentiated SH-SY5Y cells in response to Oxotremorine-M (Oxo, O100 Sigma-Aldrich) treatment was assessed in dose–effect experiments by exposing cells for 24 h to different Oxo concentrations, ranging between 1 µM and 100 µM. Time–course modulation of HSP70 expression was assessed by treating cells with Oxo (10 µM) for 24 h, 48 h, and 72 h. The impact of KNK437 treatment on Oxo-induced HSP70 expression was investigated by pre-treating cells, at day 9 of differentiation, for 24 h with KNK437 (50 µM) before exposing them to Oxo (10 µM, 24 h).

Dose-dependent response of fully differentiated SH-SY5Y cells to Tertiary-butyl hydroperoxide (TBH, 458139 Sigma-Aldrich)-induced death was assessed following 24 h exposure to different TBH doses (50 µM, 100 µM, and 150 µM).

Oxo neuroprotective and antioxidant effects were assessed in cells co-treated with TBH (150 µM) and Oxo (10 µM) for 24 h.

Finally, HSP70 involvement in Oxo neuroprotective effects in fully differentiated cells was assessed by pre-treating cells at day 9 of differentiation for 24 h with KNK437 (50 µM) before exposing them to TBH (150 µM) and Oxo (10 µM) for the following 24 h, without removing KNK437 from the culture medium. MTT test was performed on day 11 of differentiation.

In all the experiments, the control (Ctrl) groups received an equal volume of the solvent only.

### 2.3. Morphological Analysis

Cells were grown at a density of 3 × 10^4^ on glass coverslips 12 mm diameter round. Cells were fixed with 4% formaldehyde solution for 15 min at room temperature, washed twice with PBS 1× and the coverslips were mounted on slides. Representative bright field images (20×) were obtained using a Leica DMIL Led Inverted microscope equipped with a Leica ICC50 HD camera.

### 2.4. Immunocytochemistry

Cells were grown at a density of 6 × 10^4^ on glass coverslips 12 mm diameter round. Cells were fixed in their undifferentiated state or 11 days after differentiation with 4% formaldehyde solution for 15 min at room temperature. After two washes with PBS 1×, cells were pre-incubated in blocking solution (BSA 5 mg/mL and Triton 0.1% in PBS 1×) for 15 min and incubated overnight with anti-Microtubule-associated protein 2 (MAP-2) monoclonal antibody 1:400 (M4403, Sigma-Aldrich, St. Louis, MO, USA) diluted in blocking solution. After two washing with PBS 1x, fluorescent signal was visualized following 1 h incubation with a rhodamine-conjugated anti-mouse IgG Cy3 antibody 1:150 (115–165–003, Jackson ImmunoResearch). After two washes with PBS 1×, the coverslips were mounted on slides and examined, using the 20 × objective, under a fluorescence microscope (DMRBE, Leica Microsystems) equipped with digital video camera (Spot-RT Slider, Diagnostic Instruments, Sterling Heights, MI, USA). The images were adjusted for brightness and contrast with the camera software (SPOT Advanced software, v. 4.0.9, Diagnostic Instruments, Sterling Heights, MI, USA).

### 2.5. Cell Viability by MTT Test

Undifferentiated and differentiated SH-SY5Y cells were grown at a density of 2 × 10^4^ and 1.4 × 10^4^ cells/well, respectively, on 96-well plates in a final volume of 100 μL/well. Cell viability was assessed by measuring the amount of purple formazan following the reduction of 3-(4,5-dimethylthiazol-2-yl)-2,5-diphenyltetrazolium bromide (MTT, 0.5 mg/mL, Sigma-Aldrich) by viable cells after 3 h incubation at 37 °C. Absorbance was measured at 570 nm with background subtraction using Thermo Scientific^TM^ Multiskan^TM^ GO Microplate Spectrophotometer after dissolving formazan crystals with DMSO 100 μL/well.

### 2.6. Viable Cell Count by Trypan Blue Exclusion Method

Cells were grown at a density of 2 × 10^5^ cells/well on 24-well plates in a final volume of 500 μL/well. Following the indicated treatments, 10 μL of cell suspension was mixed with 90 μL of Trypan Blue (0.4% *w/v* solution, T8154 Sigma-Aldrich). 10 µL of the resulting solution was pipetted in the hemocytometer chamber for viable cell counting.

### 2.7. Western Blotting

Undifferentiated and differentiated SH-SY5Y cells were grown at a density of 2 × 10^5^ and 8 × 10^4^ cells/well, respectively, on 12-well plates in a final volume of 1 mL/well. Cells were homogenized in cold radioimmunoprecipitation assay (RIPA) buffer (50 mM Tris, pH 7.4, 150 mM NaCl, 1% Triton, SDS 0.1%), supplemented with protease inhibitor cocktail (Sigma-Aldrich P8340) and phosphatase inhibitor cocktail (Sigma-Aldrich P5726). Samples were sonicated (30 pulsations/min), quantified by the Lowry method [23] and stored at −80 °C. Western blotting was performed as previously described in [24]. Protein samples (30 µg per lane) and molecular weight marker (PageRuler^TM^ Plus Prestained Protein Ladder, 26619 ThermoFisher Scientific) were run on 8% polyacrylamide gel and electrophoretically transferred onto nitrocellulose membrane (RPN303E, Hybond-C-extra, GE Healthcare Europe GmbH). The membranes were incubated for 1 h in blocking buffer (1× TBS, 0.1% Tween−20, 5% *w/v* nonfat dry milk) and incubated with gentle shaking overnight at −4 °C with specific antibodies in blocking buffer. For the detection of HSPs, SOD1, SOD2, and pHSF1, the following antibodies were used: mouse anti-Hsp70/HSC70 (sc-24 Santa Cruz Biotechnology), mouse anti-Hsp90 α/β (sc-13119 Santa Cruz Biotechnology), rabbit anti-Hsp60 (SC-13966 Santa Cruz Biotechnology), rabbit anti-SOD1 (SC-11407 Santa Cruz Biotechnology), mouse anti-SOD2 (sc-137254 Santa Cruz Biotechnology), rabbit anti-pHSF1 (bs-3741R Bioss antibodies). The day after, membranes were washed three times for 10 min with TBS/T and incubated for 1 h at room temperature with goat anti-mouse IgG (sc-2005 Santa Cruz Biotechnology) and goat anti-rabbit IgG (sc-2357 Santa Cruz Biotechnology) horseradish peroxidase-conjugated diluted 1:10,000. For normalization of the HSP signal, membranes were exposed to horseradish peroxidase-conjugated β-Actin primary antibody (sc-2005 Santa Cruz Biotechnology), diluted 1:10,000, for 1 h. After three washings with TBS-T, immunocomplexes were visualized with chemiluminescence reagent (SuperSignal^TM^ West Pico PLUS, ThermoFisher Scientific). The membranes were exposed to autoradiography film (Amersham Hyperfylm ECL, 28-9068-36). Chemiluminescent signal was visualized and fixed in Kodak D19 developer and fixer (1900984 and 1902485, Kodak GBX). A sample of chemiluminescent membranes was developed by iBright FL1000 Imaging System (ThermoFisher Scientific). The densitometric evaluation of bands was performed by measuring the optical density (O.D.) using NIH ImageJ software. Original Western blotting images of biological replicates used for the quantitative analysis have been included in the Supplementary Materials.

### 2.8. ROS Quantification

To assess ROS generation, SH-SY5Y differentiated cells were grown at a density of 1.4 × 10^4^ cells/well on 96-well plates in a final volume of 100 μL/well. At the end of treatments, cells were incubated with 1 mM dichlorofluorescein diacetate (DCFH-DA, 35845 Sigma-Aldrich) for 10 min in the dark at room temperature. The conversion of the non-fluorescent cell-permeant DCFH-DA to the green fluorescent compound 20, 70-dichlorofluorescein (DCF) by esterase activity is used as an indicator of intracellular ROS levels. The fluorescence was measured by using a microplate Reader GloMax fluorimeter (Promega Corporation) at the excitation wavelength of 475 nm and emission wavelength of 555 nm.

Fluorescent representative morphological pictures were captured by using the fluorescence Zeiss Axio Scope 2 microscope (Carl Zeiss, Oberkochen, Germany). Cell nuclei were visualized by Hoechst staining.

### 2.9. Statistical Analysis

Data for statistical analysis were collected from at least three independent experiments. Data analysis was performed using GraphPad Prism 8.4.3. software (GraphPad Software, Inc., La Jolla, CA, USA). Quantification of optical density of Western blotting bands was expressed as arbitrary units, with controls equal to 1. Quantification of cell viability and DCF signal (ROS levels) was expressed as percentage, with control group equal to 100%. Normal distribution of data was assessed by Shapiro–Wilk test. For data normally distributed, statistical evaluations were performed by one-way ANOVA, followed by Tukey Post-Hoc test. *t*-test was used for the statistical comparison between the means of two groups. The relative results were presented as mean ± SD. For data not normally distributed, statistical evaluations were performed by Kruskal–Wallis test, followed by Dunn’s multiple comparison test. The relative results were displayed as median with interquartile range. Differences in *p*-value less than 0.05 were considered statistically significant.

## 3. Results

### 3.1. SH-SY5Y Differentiation

**Figure 1:**
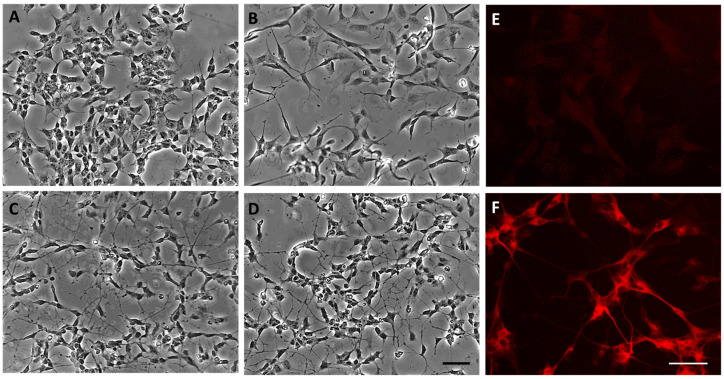
Differentiation of SH-SY5Y cells. Phase-contrast pictures of undifferentiated cells (**A**), cells differentiated for 4 days (**B**), 8 days (**C**), and 11 days (**D**). MAP-2 fluorescent staining in SH-SY5Y cells undifferentiated (**E**) and differentiated for 11 days (**F**). Scale bar: 50 µm.

The protocol of cell differentiation was initiated 24 h after plating and was completed within 11 days. Undifferentiated cells, characterized by a flat morphology, display non-polarized cell bodies and short neurite projections (Figure 1A). As the process of differentiation progresses, cells stop proliferating and gradually develop long and branched neurites (Figure 1B–D). We considered SH-SY5Y cells fully differentiated after 11 days, when the complex network of neurite projections was clearly visible (Figure 1D). This morphological maturation is associated with the increased expression of MAP-2 (Figure 1E,F).

### 3.2. Modulation of HSP Expression during SH-SY5Y Differentiation

**Figure 2:**
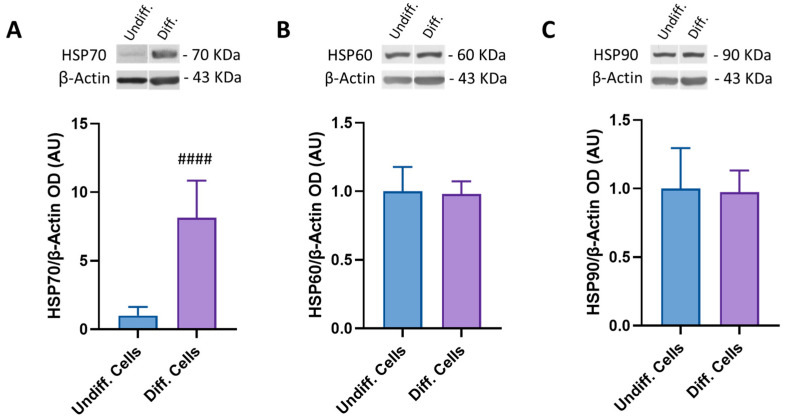
HSP expression in undifferentiated (Undiff.) and fully differentiated (Diff., 11 days) cells. Representative Western blotting band images and histogram of (**A**) HSP70, (**B**) HSP60, and (**C**) HSP90 normalized to β-Actin optical density (OD). *t*-test #### *p* < 0.0001 as compared to Undiff. cell group. AU (Arbitrary Units).

Since the differentiation process is considered a stressful event associated with significant changes in cell metabolism and survival, we first checked the expression of different HSPs, involved in maintaining cellular homeostasis during stress conditions. By comparing the protein levels of HSP60, HSP70, and HSP90 in undifferentiated and fully differentiated SH-SY5Y cells (11 days), we found that HSP70 was dramatically increased in differentiated SH-SY5Y cells (Figure 2A), while no significant modulation was observed for HSP60 (Figure 2B) and HSP90 (Figure 2C).

### 3.3. HSP70 Is Involved in SH-SY5Y Cell Survival during the Differentiation Process

**Figure 3:**
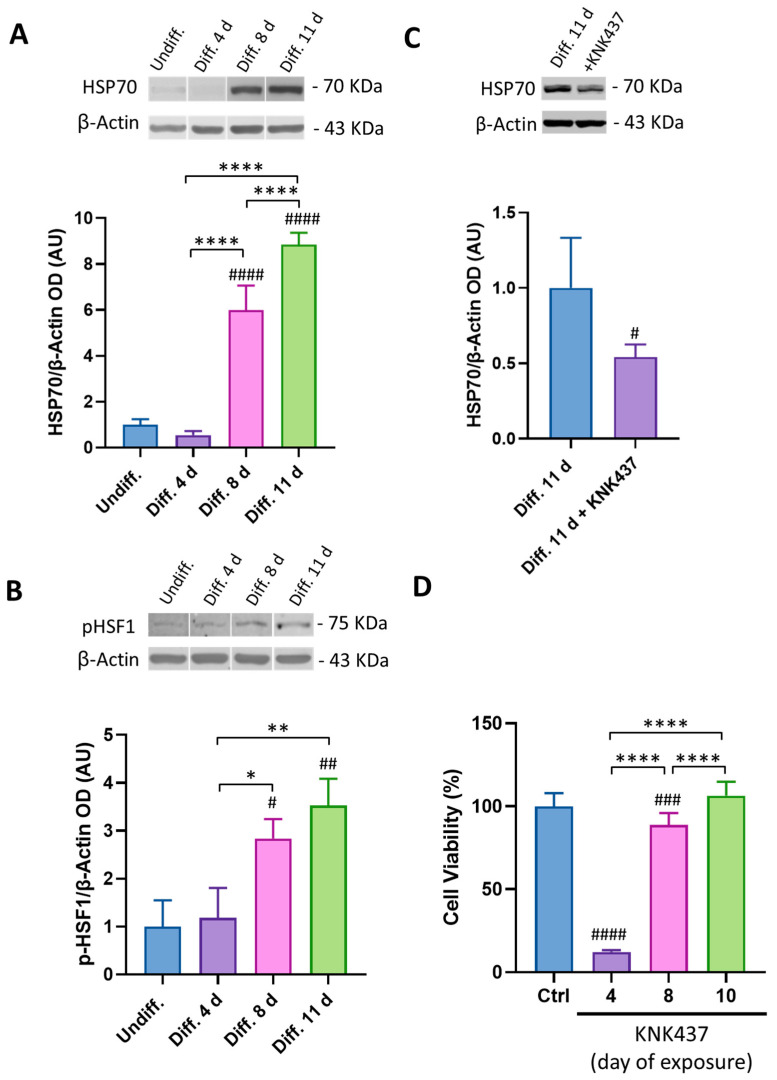
HSP70 and pHSF1 expression and function in SH-SY5Y differentiation. (**A**) Representative images of HSP70 and β-Actin Western blotting bands and histogram of HSP70 normalized to β-Actin optical density (OD) in undifferentiated (Undiff.) cells and cells differentiated (Diff.) for 4, 8, and 11 days. (**B**) Representative images of phosphorylated HSF1 (pHSF1) and β-Actin Western blotting bands and histogram of p-HSF1 normalized to β-Actin OD in undifferentiated (Undiff.) cells and cells differentiated (Diff.) for 4, 8, and 11 days. (**C**) Representative images of HSP70 and β-Actin Western blotting bands and histogram of HSP70 normalized to β-Actin OD in non-treated fully differentiated (Diff.) cells (11 days) and in fully differentiated cells treated at day 8 with KNK437 (50 µM). (**D**) Cell viability of fully differentiated cells (11 days) treated with KNK437 (50 µM) at day of differentiation 4, 8, and 10, evaluated by MTT test. Tukey test: # *p* < 0.05, ## *p* < 0.01, ### *p* < 0.001, #### *p* < 0.0001 as compared to Undiff. Cells in (**A**,**B**), or to Ctrl group in (**D**); * *p* < 0.05, ** *p* < 0.01, **** *p* < 0.0001 in (**A**,**B**,**D**); *t*-test in (**C**) # *p* < 0.05. AU (Arbitrary Units).

In order to explore whether HSP70 may play a role in the differentiation of SH-SY5Y cells, we first checked its expression during the entire duration of the process. Surprisingly, the basal levels of HSP70 protein observed in undifferentiated cells were further decreased in cells differentiated for 4 days, while an exponential increase in HSP70 levels was observed in cells differentiated for 8 and 11 days (Figure 3A).

In the attempt to investigate the putative mechanism involved in the modulation of HSP70 expression, we focused our attention on the activation of the Heat shock factor 1 (HSF1) protein, the primary modulator of the heat shock response (HSR) under stress condition [25]. In line with the expression of HSP70 during the differentiation process, the phosphorylation of HSF-1 (p-HSF1) was significantly increased in cells differentiated for 8 and 11 days, as compared with undifferentiated cells (Figure 3B).

We next assessed the impact of HSP70 expression inhibition by KNK437 (50 µM, Figure 3C) on SH-SY5Y cell survival during the differentiation process. In particular, we assessed the cell viability of fully differentiated SH-SY5Y cells (11 days) following the exposure to KNK437 for 1 day (applied at day 10 of differentiation), 3 days (applied at day 8 of differentiation), and 7 days (applied at day 4 of differentiation). Interestingly, the results of the MTT test, supported by visual inspection and the count of viable cells by the Trypan Blue exclusion method (Figure S1A), revealed that inhibition of HSP70 expression initiated on the fourth day of differentiation causes a dramatic increase in cell death (Figure 3D). This information clearly suggests that HSP70 is involved in maintaining cell survival during the process of differentiation. Similar results were obtained when the MTT test was carried on at day 7 of differentiation, following exposure to KNK437 for 3 days (Figure S1B). On the contrary, if KNK437 treatment was initiated later when HSP70 was already expressed enough to ensure cell survival, cell death was strongly reduced or even absent (Figure 3D).

### 3.4. HSP70 Is Involved in SH-SY5Y Cell Susceptibility to H_2_O_2_-Induced Cell Death

**Figure 4:**
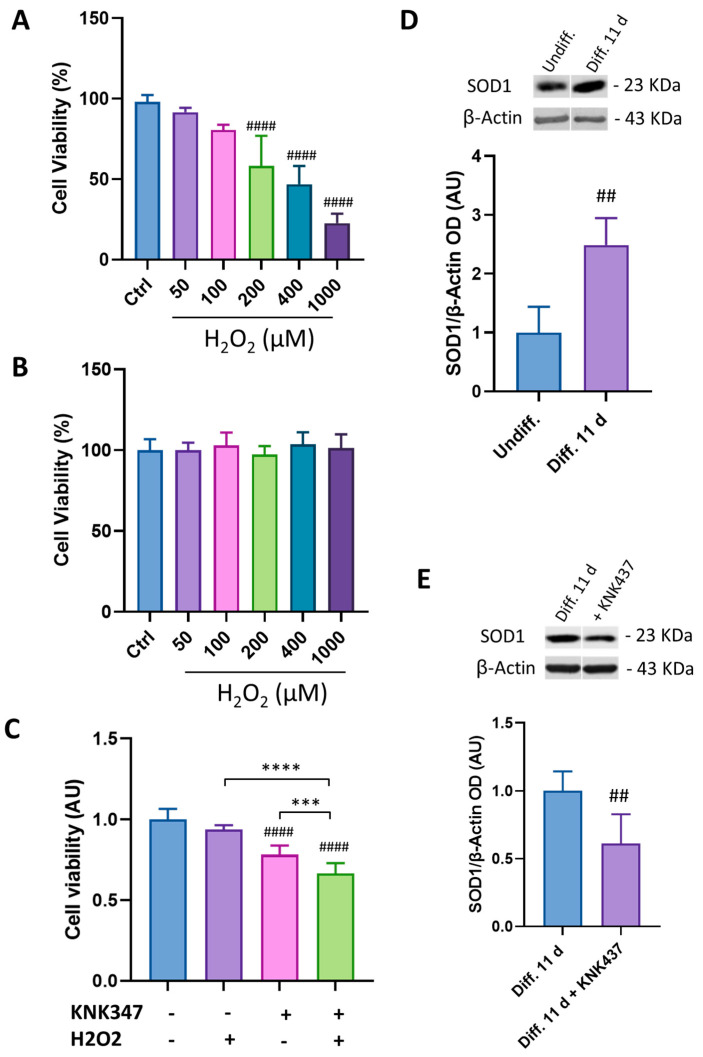
HSP70 and SOD1 role in SH-SY5Y protection against H_2_O_2_—induced cell death. (**A**) Dose–effect of H_2_O_2_ treatment (24 h) on undifferentiated cell viability. (**B**) Dose–effect of H_2_O_2_ treatment (24 h) on fully differentiated (11 days) cell viability. (**C**) Cell viability of fully differentiated cells (11 days) treated with KNK437 50 µM (at day 8 of differentiation) alone or in combination with H_2_O_2_ 200 µM (at day 10 of differentiation). (**D**) Representative images of SOD1 and β-Actin Western blotting bands and histogram of SOD1 normalized to β-Actin optical density (OD) in undifferentiated (Undiff.) cells and fully differentiated (Diff.) cells (11 days). (**E**) Representative images of SOD1 and β-Actin Western blotting bands and histogram of SOD1 normalized to β-Actin OD in non-treated fully differentiated (Diff.) cells (11 days) and in fully differentiated cells treated at day 8 with KNK437 (50 µM). Dunn’s test in (**A**) #### *p* < 0.0001 as compared to control (Ctrl) group; Tukey test in (**B**,**C**) #### *p* < 0.0001 as compared to control (Ctrl) non-treated group; *** *p* < 0.001; **** *p* < 0.0001 in (**C**); *t*-test in (**D**,**E**) ## *p* < 0.01 as compared to undifferentiated cells in (**D**), and non-treated fully differentiated cells in (**E**). AU (Arbitrary Units).

Since HSP70 is involved in the protection of cells against different stress conditions, including oxidative stress, we tested whether HSP70 expression affects SH-SY5Y cells’ response to H_2_O_2_ treatment. We first evaluated cell viability by MTT test in undifferentiated SH-SY5Y cells. As expected, exposure of undifferentiated SH-SY5Y cells to the strong oxidizing agent H_2_O_2_ induced a dose-dependent decrease in cell viability, already significant at the 200 µM dose (Figure 4A). Interestingly, the same treatment, even at higher doses, produced no significant change in SH-SY5Y differentiated cell viability (Figure 4B). However, when SH-SY5Y cells were pre-treated for 2 days (since day 8 of differentiation) with KNK437 before H_2_O_2_ exposure (at day 10 of differentiation, 200 µM dose), we observed a significant reduction in cell viability (Figure 4C). These data suggest that HSP70 expression is involved in SH-SY5Y differentiated cell protection against H_2_O_2_-induced cell death. HSP70 modulates redox homeostasis by multiple signaling pathways, including the regulation of SOD activity [17,18]. Here, we observed a significant increase in the levels of SOD1 protein in fully differentiated SH-SY5Y cells, as compared to undifferentiated cells (Figure 4D), likely involved in the protection of differentiated cells against H_2_O_2_-induced cell death. This upregulation was partially blocked when cells were pre-treated for 72 h with KNK437 (Figure 4E), demonstrating the specific involvement of HSP70 in this modulation. Surprisingly, we did not detect significant changes in SOD2 expression between undifferentiated and differentiated SH-SY5Y cells (Figure S2).

### 3.5. Oxo Induces HSP70 and pHSF1 Expression in SH-SY5Y Differentiated Cells

**Figure 5:**
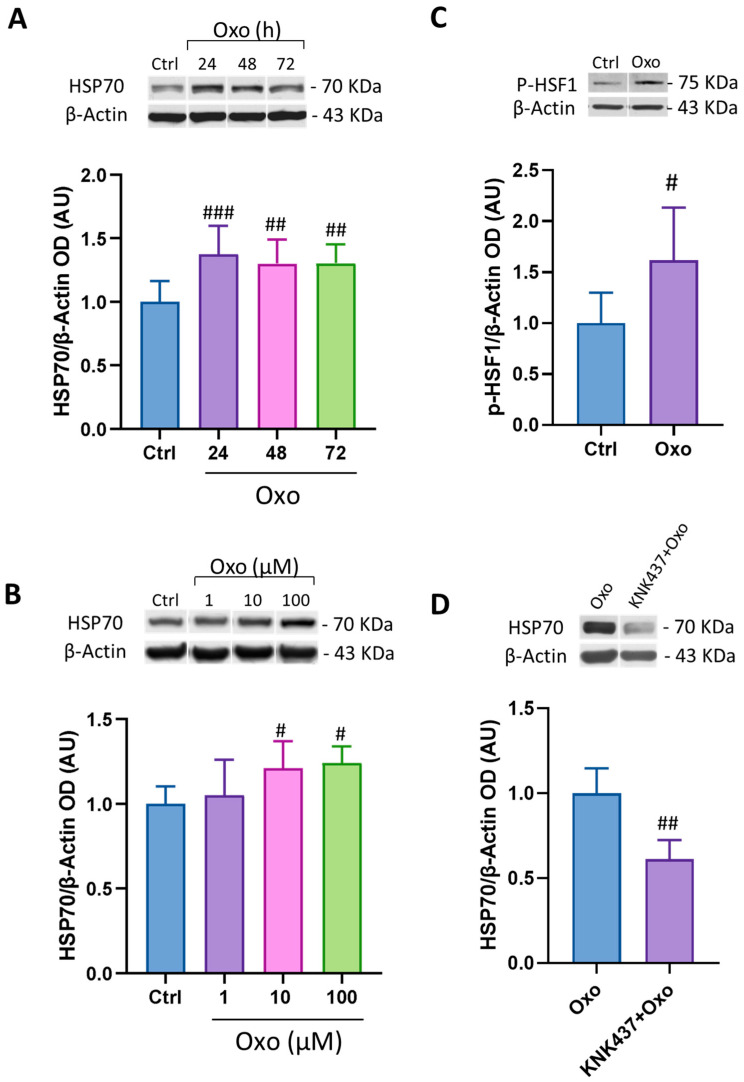
Modulation of HSP70 and pHSF1 expression by Oxo treatment. (**A**) Representative images of HSP70 and β-Actin Western blotting bands and histogram of HSP70 normalized to β-Actin optical density (OD) in time–course experiment following Oxo exposure (10 µM) in fully differentiated SH-SY5Y cells. (**B**) Representative images of HSP70 and β-Actin Western blotting bands and histogram of HSP70 normalized to β-Actin OD in dose–effect experiment following Oxo exposure (24 h). (**C**) Representative images of phosphorylated HSF1 (pHSF1) and β-Actin Western blotting bands and histogram of pHSF1 normalized to β-Actin OD in fully differentiated SH-SY5Y cells treated with Oxo (10 µM) for 24 h. (**D**) Representative images of HSP70 and β-Actin Western blotting bands and histogram of HSP70 normalized to β-Actin OD of cells pre-treated for 24 h with KNK437 (50 µM) before Oxo exposure (10 µM, 24 h). Dunn’s test in (**A**) ## *p* < 0.01, ### *p* < 0.001 as compared to Control (Ctrl) group; Tukey test in (**B**) # *p* < 0.05 as compared to Control (Ctrl) group; *t*-test in (**C**,**D**) # *p* < 0.05, ## *p* < 0.01 as compared to Ctrl group in (**C**) and Oxo-treated group in (**D**). AU (Arbitrary Units).

Since previous studies demonstrated that Oxo treatment was able to induce an increase in HSP70 expression in the rat hippocampus [21], we have now tested the modulation of HSP70 expression in fully differentiated SH-SY5Y cells in response to Oxo treatment. Time–course (Figure 5A) and dose–effect (Figure 5B) experiments showed that HSP70 levels were increased by Oxo treatment at all examined time points for doses ranging between 10 and 100 µM. By testing the effect of Oxo 10 µM, the lowest dose producing a significant increase in HSP70 expression, we found that the increase in HSP70 expression was coupled with the enhancement of p-HSF1 levels (Figure 5C). Furthermore, pre-treatment with KNK437 abolished the Oxo-induced increase in HSP70 levels (Figure 5D).

### 3.6. Oxo Treatment Rescues Cell Viability and Oxidative Stress Induced by Cell Exposure to TBH by Enhancing HSP70 and SOD1 Expression

**Figure 6:**
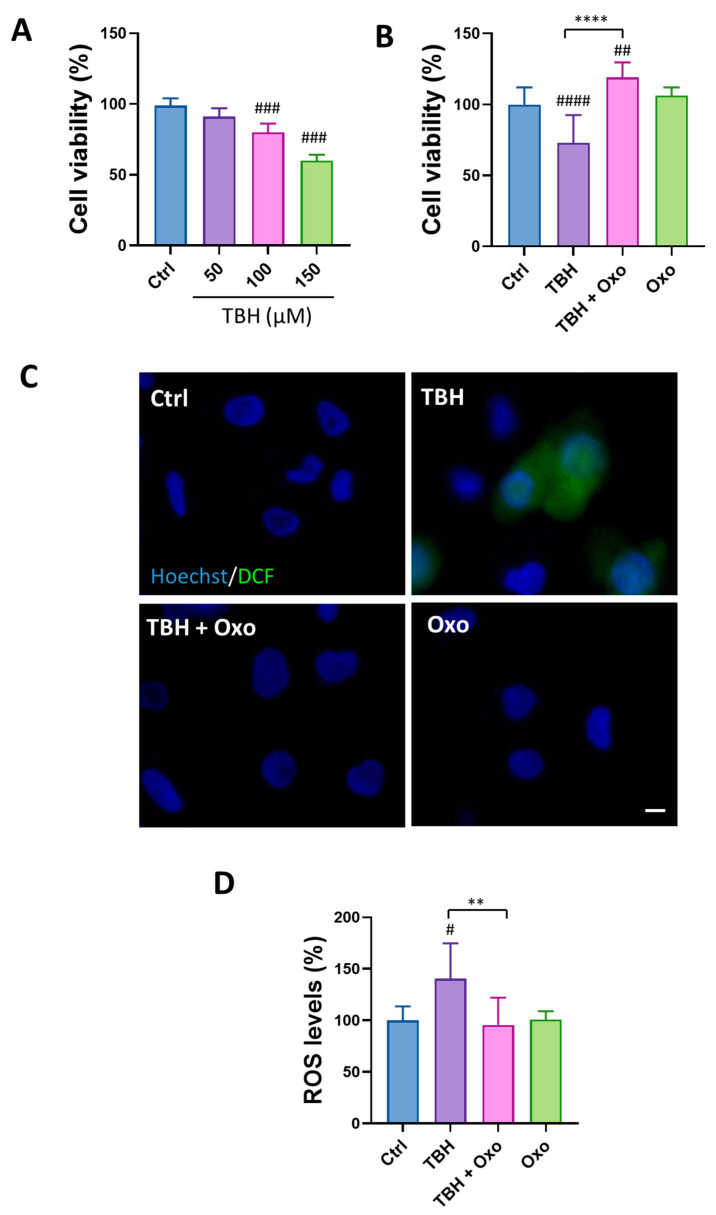
Oxo protects differentiated cells against TBH-induced oxidative stress. (**A**) Dose–effect of TBH treatment (24 h) on fully differentiated SH-SY5Y cell viability. (**B**) Quantification of cell viability in control (Ctrl) cells, cells treated with TBH (150 µM, 24 h), TBH (150 µM, 24 h) + Oxo (10 µM, 24 h), and Oxo alone (10 µM, 24 h). (**C**) Representative pictures of ROS generation, visualized by green fluorescent DCF signal. Cell nuclei are visualized by Hoechst staining. (**D**) DCF fluorescence intensity quantification, index of ROS levels. Scale bar 50 µm. Dunn’s test in (**A**) ### *p* < 0.001 as compared to Ctrl group; Tukey test in (**B**,**D**) # *p* < 0.05, ## *p* < 0.01, #### *p* < 0.0001 as compared to Ctrl group; ** *p* < 0.01, **** *p* < 0.0001.

We next tested the neuroprotective effect of Oxo treatment against cell death induced by exposure to tert–Butyl hydroperoxide (TBH), a chemical that triggers oxidative stress through catalysis by glutathione peroxidase to form oxidized glutathione and tertiary butyl alcohol [26]. Fully differentiated SH-SY5Y cells treated for 24 h with TBH showed a dose-dependent significant decrease in cell viability, as assessed by the MTT test (Figure 6A). TBH 150 μM was chosen for the subsequent investigations. Oxo treatment (10 µM, 24 h), which per se did not induce any significant change in cell viability, was able to increase cell survival impaired by TBH treatment significantly and to restore neuronal viability fully (Figure 6B). Since the neurotoxic effect of TBH is linked to the generation of oxidative stress, we assessed the impact of Oxo on TBH-induced ROS production by Dichloro-dihydro-fluorescein diacetate (DCFH-DA) fluorescence intensity assay. Visualization and quantification of 20, 70-dichlorofluorescein (DCF) fluorescence intensity and index of ROS generation showed that treatment of SH-SY5Y differentiated cells with Oxo completely elapsed ROS increase driven by TBH exposure (Figure 6C,D).

**Figure 7:**
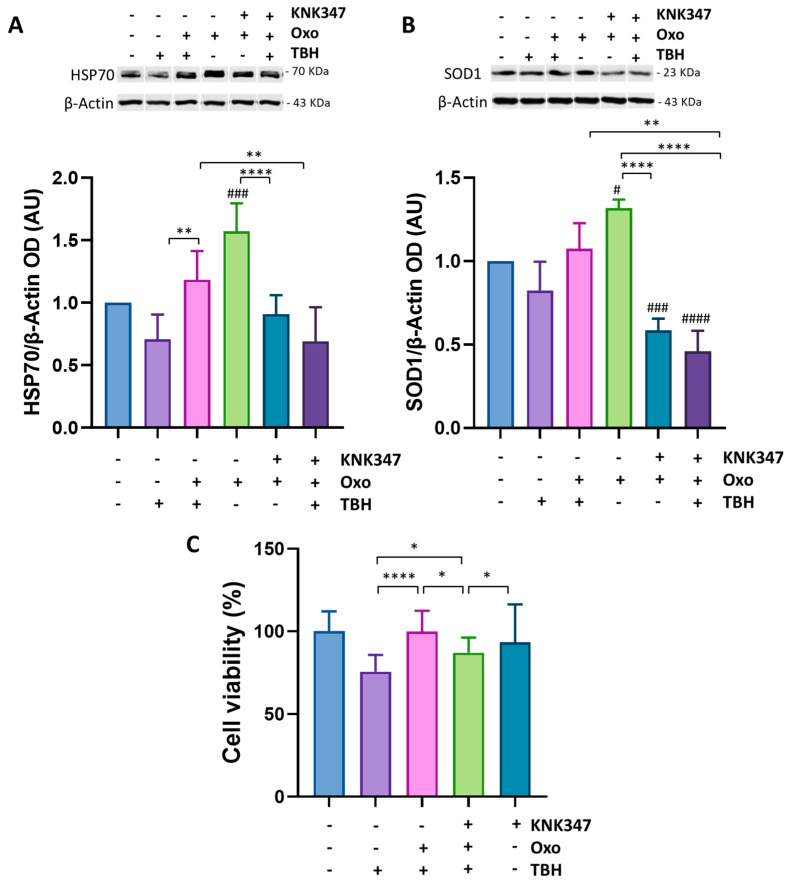
Role of HSP70 and SOD1 in Oxo-mediated neuroprotective effects. (**A**) Representative images of HSP70 and β-Actin Western blotting bands and histogram of HSP70 normalized to β-Actin optical density (OD) in untreated control (Ctrl) cells, cells treated with TBH (150 µM for 24 h), TBH (150 µM for 24 h) + Oxo (10 µM for 24 h), TBH (150 µM for 24 h) + Oxo (10 µM for 24 h) + KNK437 (50 µM for 48 h), Oxo (10 µM for 24 h), and Oxo (10 µM for 24 h) + KNK437 (50 µM for 48 h). (**B**) Representative images of SOD1 and β-Actin Western blotting bands and histogram of SOD1 normalized to β-Actin OD in untreated control (Ctrl) cells, cells treated with TBH (150 µM for 24 h), TBH (150 µM for 24 h) + Oxo (10 µM for 24 h), TBH (150 µM for 24 h) + Oxo (10 µM for 24 h) + KNK437 (50 µM for 48 h), Oxo (10 µM for 24 h), and Oxo (10 µM for 24 h) + KNK437 (50 µM for 48 h). (**C**) Cell viability quantification in control (Ctrl) cells, cells treated with TBH (150 µM for 24 h), TBH (150 µM for 24 h) + Oxo (10 µM for 24 h), TBH (150 µM for 24 h) + Oxo (10 µM for 24 h) + KNK437 (50 µM for 48 h), KNK437 (50 µM for 48 h). Tukey test # *p* < 0.05, ### *p* < 0.001, #### *p* < 0.0001 as compared to Ctrl non-treated group; * *p* < 0.05, ** *p* < 0.01, **** *p* < 0.0001. AU (Arbitrary Units).

Next, in order to assess whether HSP70/SOD1 signaling was involved in Oxo antioxidant and protective effects, cells received treatment with KNK437 (50 µM) 24 h before Oxo (10 µM) and TBH (100 µM) exposure (24 h). Notably, both HSP70 and SOD were increased by Oxo treatment in TBH-exposed cells, while their expression was significantly inhibited by KNK437 (Figure 7A,B). Finally, co-treatment with KNK437 reduced Oxo-induced protection against oxidative stress damage (Figure 7C), suggesting the involvement of HSP70/SOD1 in this effect.

## 4. Discussion

In this study, we demonstrated that manipulation of the HSP70 signal modulates SH-SY5Y cell differentiation and susceptibility to oxidative stress-dependent cell death. Given their property to differentiate into a neuronal-like phenotype, human neuroblastoma SH-SY5Y cells are broadly exploited for screening projects in neuroscience. Several differentiation protocols have been established in order to optimize the overall properties of SH-SY5Y as a reliable neuronal cell culture model or to enhance a specific neuronal phenotype. RA, employed in most of the differentiation protocols, down-regulates the mRNA and protein levels of the differentiation-inhibiting basic helix–loop–helix transcription factors [27]. Moreover, RA activates survival signaling in SH-SY5Y cells [28,29] and drives the differentiation toward the cholinergic/dopaminergic neuronal phenotype by inducing the increase in the expression of Acetylcholine transferase and vesicular monoamine transporter [8,30,31,32]. In this study, RA was used to induce the differentiation of SH-SY5Y cells, in combination with serum deprivation and the addition of Neurobasal medium and B27 supplement, which have been shown to enhance cell differentiation [2]. By following the modifications in cell proliferation rate and morphology, we found that SH-SY5Y is fully differentiated 11 days following the beginning of the differentiation protocol, showing a decrease in the proliferation rate and a visible network of branched neurite projections. However, despite the use of B27 supplement and neurobasal medium, many cells do not survive this process, which has been considered a stressful event [4]. Cells can trigger different responses to stress conditions, including the enhancement of pro-survival pathways and the activation of cell death to eventually remove injured cells [33]. One of the main pro-survival activities of cells under stress conditions is represented by the heat shock response involving HSPs. Even though some HSPs are constitutively produced, most of them are usually over-expressed by cells in response to internal and external stress signals that may cause protein denaturation, including heat, nutrient deficiency, oxidative stress, inflammation, and microbial infections [34]. In this study, we found that the differentiation process drives the expression of HSP70 without affecting HSP60 and HSP90 levels. In line with our results, other investigations, by using a protocol of differentiation with both RA and BDNF supplements lasting 7 days, have already described the increase of HSP70 expression in neuron-like SH-SY5Y cells; this enhancement of HSP70 levels was responsible for protection against staurosporine-induced apoptosis [35] and hyperthermic stress [12]. Intriguingly, we found an initial not statistically significant downregulation of HSP70 after 4 days of differentiation, likely related to the inhibition of proliferation and tumorigenicity in the early phase of differentiation [36,37]. This downregulation was followed by an exponential increase of HSP70 expression at 8 and 11 days of differentiation, probably involved in the survival of differentiated cells. HSP70 plays a key role in maintaining proteome integrity and cell viability under different stress conditions, including serum deprivation [38], and some data also suggest its involvement in neuronal development [39], including neuroblast differentiation in the hippocampus [40]. Accordingly, we found that inhibition of HSP70 synthesis at the critical window of induction of HSP70 expression during the differentiation causes a dramatic increase in SH-SY5Y cell death, clearly indicating that this chaperone is involved in guaranteeing cell survival. Interestingly, the modulation of HSP70 expression during the differentiation of SH-SY5Y cells is accompanied by the phosphorylation of HSF1, a central regulator of the heat shock response, which establishes a complex bidirectional feedback loop with HSP70 expression [41]. Indeed, on the one hand, HSF1 is inhibited by chaperone interactions; on the other, during proteostasis perturbations, chaperone availability decreases as a result of sequestration by misfolded proteins, and the subsequent activation of HSF1 upregulates the expression of key proteostasis genes, including chaperones [25]. Although in our experimental model, HSP60 and HSP90 expression are not modulated by the differentiation process, we cannot exclude that other factors and chaperones, including HSP27, may be involved in the survival of differentiated SH-SY5Y cells.

HSP70 also participates in maintaining redox homeostasis [42]. “Oxidative stress” indicates a pathological condition characterized by the imbalance between oxidants and antioxidants in favor of the oxidants, which leads to the impairment of redox control and subsequent cellular damage. Normally, the cell-reducing internal environment plays a major role in antioxidant defenses; however, the excess of free radicals inside cells can saturate the antioxidant defenses, leading to oxidative macromolecular damage and cell death [43].

Oxidative stress is a leading mechanism in neurological disorders [44]. Indeed, oxidative damage develops early, in the initial phase of the disease, and prior to the full manifestation of the pathology, thus suggesting that oxidative imbalance represents a crucial trigger of the pathogenesis of neurodegenerative diseases [45].

HSP70 can protect from oxidative stress damage directed to proteins by inhibiting their aggregation and/or enhancing the degradation of oxidized proteins; moreover, HSP70 increases the expression and the activity of vital antioxidant enzymes, including SOD and catalase, and antioxidant intracellular signaling pathways [42,46]. Here, we found that differentiated SH-SY5Y cells, which express higher levels of HSP70, are not susceptible to H_2_O_2_-induced cell death, as compared to undifferentiated cells. In line with our results, previous studies demonstrated that SH-SY5Y cells, differentiated for 5 days with RA, are substantially more resistant to cytotoxicity and mitochondrial dysfunction induced by oxidative stress due to changes in mitochondrial metabolism and antioxidant defenses [47]. In contrast with these observations, another study reported that SH-SY5Y cells differentiated for 4 days with RA in a medium containing 10% FBS, showing increased sensitivity to H_2_O_2_-induced cell death due to the impairment of heme oxygenase-1 (HO-1) activation [48]; similarly, SH-SY5Y cells differentiated for 6 days following a two-step protocol that employs RA in the first phase and Neurobasal medium supplemented with N-2 and BDNF in the second one display reduced mitochondrial membrane potential and increased sensitivity to perturbations induced by 6-hydroxydopamine [49]. Overall, these observations clearly suggest that the differentiation protocol strongly affects the phenotype of differentiated cells, their protein expression, and the subsequent response to oxidative stress conditions. Interestingly, the different expression of HSP70 in undifferentiated and differentiated SH-SY5Y cells seems to affect SOD1 expression and the subsequent susceptibility to oxidative stress-dependent cell death. Indeed, when the synthesis of HSP70 is inhibited by KNK437, SOD1 expression is significantly reduced, and differentiated SH-SY5Y cells become partially susceptible to H_2_O_2_-induced cell death. Although these results support an important role for HSP70/SOD1 in influencing SH-SY5Y cell susceptibility to H_2_O_2_-induced cell death, they also suggest the involvement of other mechanisms, including changes in bioenergetics and other antioxidant defenses induced by the differentiation process [47]. Surprisingly, in our experimental model, we detected low levels of SOD2 expression, in both undifferentiated and differentiated SH-SY5Y cells, in contrast with published data reporting the increase in SOD2 levels in SH-SY5Y cells differentiated for 5 days with RA [47]. We hypothesized that the differentiation protocol or, more likely, the protocol of protein extraction, not specifically targeted to isolate mitochondrial proteins, can explain this difference.

Activation of mAchRs is responsible for the induction of several biochemical cascades [50,51,52] and neuroprotective effects [53,54]. We have recently demonstrated that Oxo, a mAchR agonist, is able to upregulate HSPs in the rat hippocampus [21]. Here we found that Oxo treatment increases HSP70 and p-HSF1 levels in SH-SY5Y differentiated cells and decreases ROS production and cell death induced by TBH exposure. These results are supported by previous findings reporting the ability of Oxo treatment to significantly decrease ROS levels in the rat hippocampus [55] and in SH-SY5Y cells exposed to Aβ_1–42_ [56]. Moreover, as further confirmation, treatment with scopolamine (a mAchRs antagonist) or M1 receptor deficiency produce an increase in oxidative stress [57,58,59], while treatment with Xanomeline, a mAchR agonist, protects cortical cells from oxygen-glucose deprivation by inhibiting oxidative stress and apoptosis [60]. Interestingly, here we found that inhibition of HSP70 and the subsequent SOD1 expression by KNK437 significantly reduced Oxo neuroprotective effects, clearly suggesting the involvement of these proteins in this beneficial outcome. As expected, inhibition of HSP70 expression does not completely abolish Oxo-induced neuroprotection, arguing that other HSP70-independent mechanisms, including the enhancement of mitochondria functionality and metabolism [56], may contribute to the observed effects.

In conclusion, our study demonstrates that the manipulation of HSP70/SOD1 expression modulates SH-SY5Y cell differentiation and susceptibility to oxidative stress-dependent cell damage, encouraging further studies aimed to better characterize HSP70’s pivotal role in driving neuronal differentiation and protection in oxidative stress-related disorders. Moreover, our data unravel novel mechanisms underlying Oxo’s neuroprotective function, suggesting novel targets for neurodegenerative disease therapy.

## Data Availability

The data that support the findings of this study are available on reasonable request.

## Supplementary Materials

The following supporting information can be downloaded at: https://www.mdpi.com/article/10.3390/antiox12030687/s1, Figure S1: Effects of KNK437 treatment on SH-SY5Y viability; Figure S2: SOD2 expression in SH-SY5Y differentiation. Original western blotting images.
