# Supplementary material for: Manipulation of HSP70-SOD1 Expression Modulates SH-SY5Y Differentiation and Susceptibility to Oxidative Stress-Dependent Cell Damage: Involvement in Oxotremorine-M-Mediated Neuroprotective Effects
Source: Antioxidants (Basel). 2023 Mar 10;12(3):687. doi: 10.3390/antiox12030687 (PMC10045076; doi:10.3390/antiox12030687)

SUPPLEMENTARY INFORMATION

Figure S1

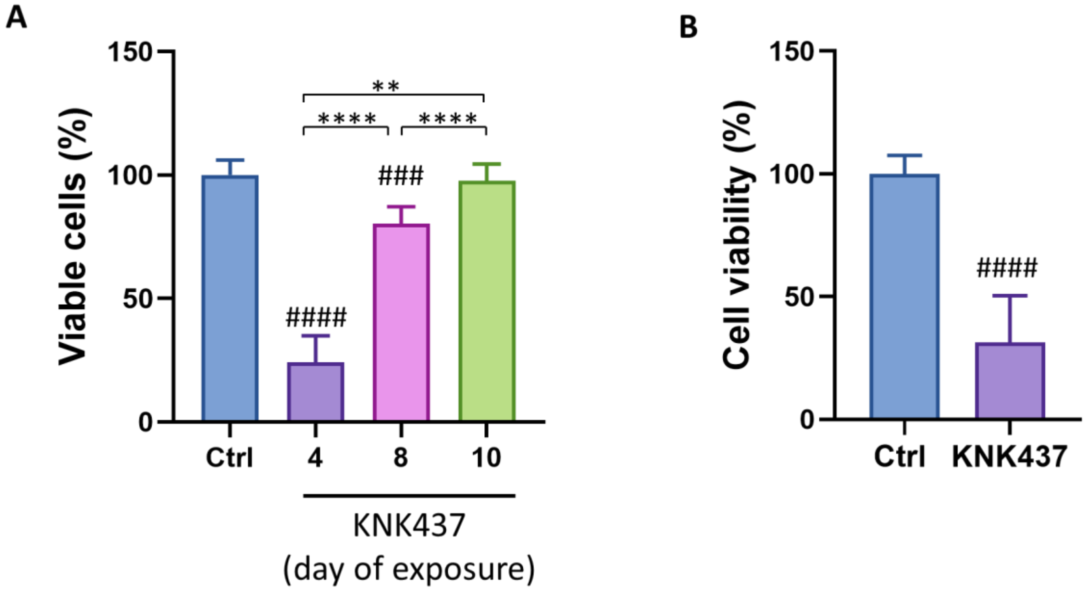

Figure S1: Effects of KNK437 treatment on SH-SY5Y viability. A) Cell viability of fully differentiated cells (11 days) treated with KNK437 (50 µM) at day of differentiation 4, 8 and 10, evaluated by Trypan Blue exclusion method. B) Viability of cells differentiated for 7 days treated with KNK437 (50 µM) at day of differentiation 4, evaluated by MTT test. Tukey test in A): ###p < 0.001, #### p < 0.0001 as compared to control (Ctrl) non-treated group, ** p < 0.01, **** p < 0.0001; t-test in B) #### p < 0.0001 as compared to Ctrl non-treated group.

Figure S2

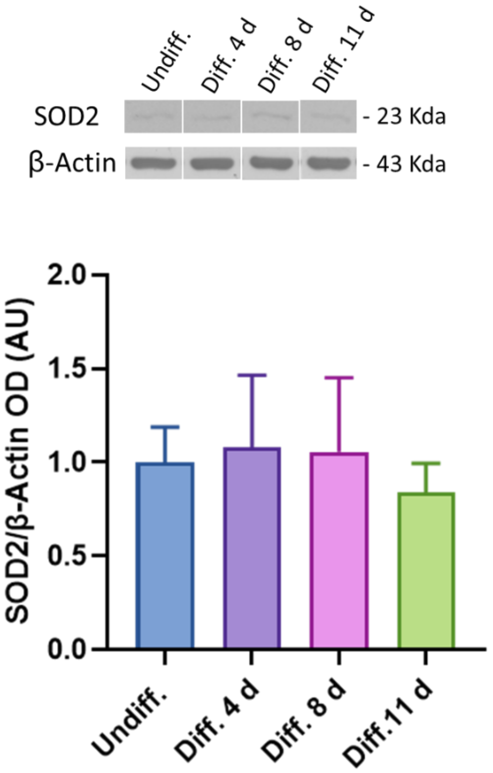

Figure S2: SOD2 expression in SH-SY5Y differentiation. Representative images of SOD2 and β-Actin western blotting bands and histogram of SOD2 normalized to β-Actin optical density (OD) in undifferentiated (Undiff.) cells and cells differentiated (Diff.) for 4, 8 and 11 days. Tukey test: $p>0.05$.

Original western blotting images of biological replicates used for statistical analysis. Arrows indicate the bands selected for the representative images shown in the manuscript.

Figure 2 A-B

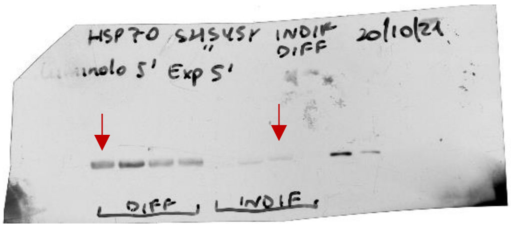

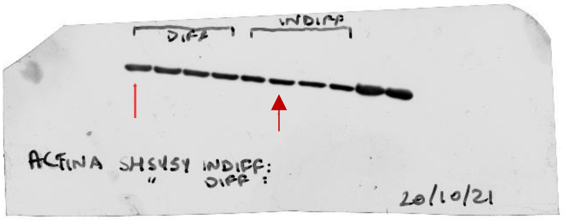

Figure 2C-D

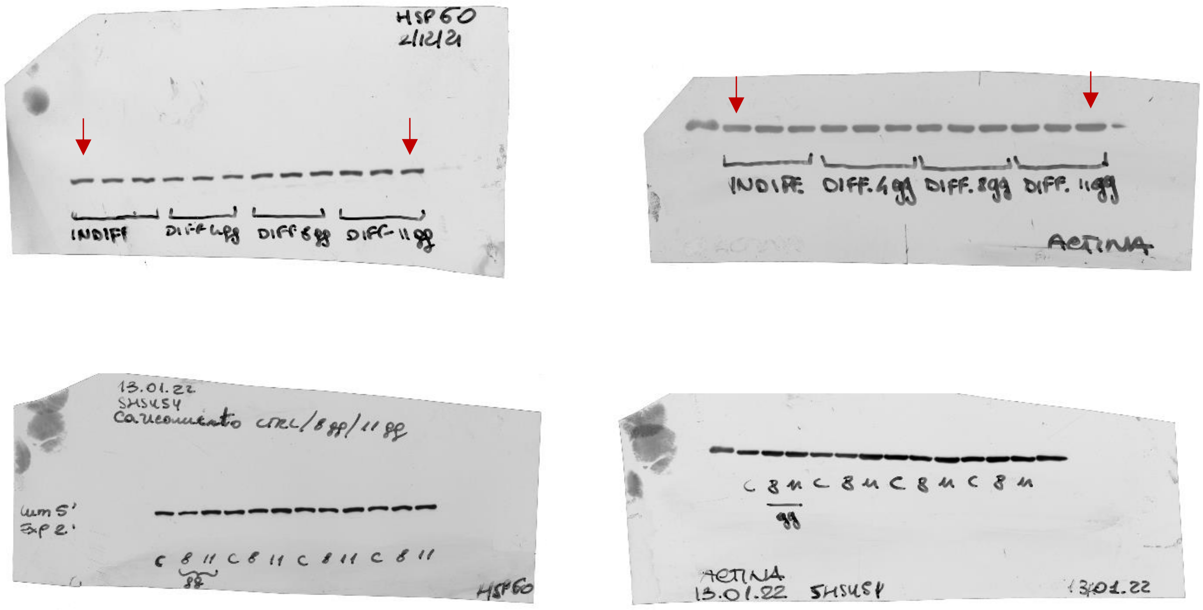

Figure 2E-F

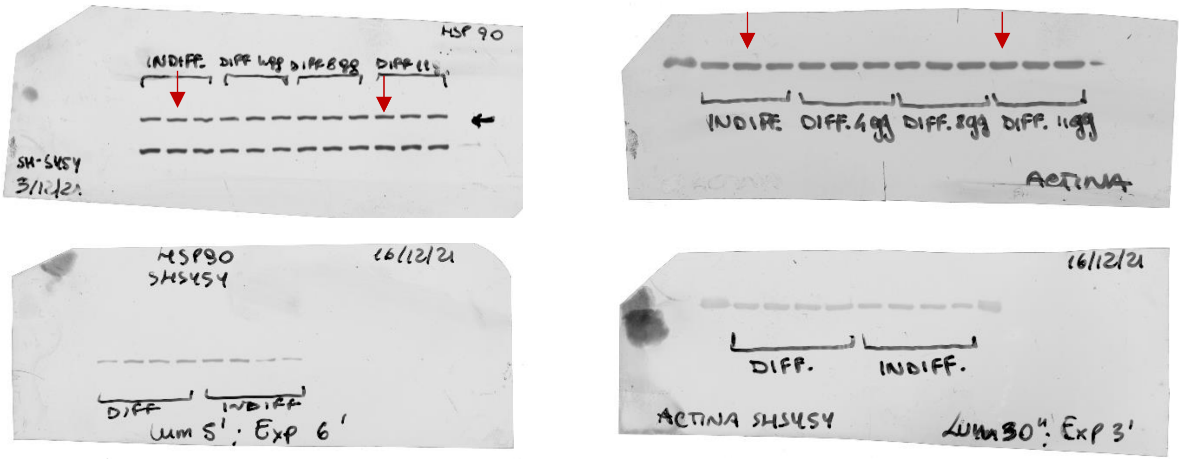

Figure 3A-B

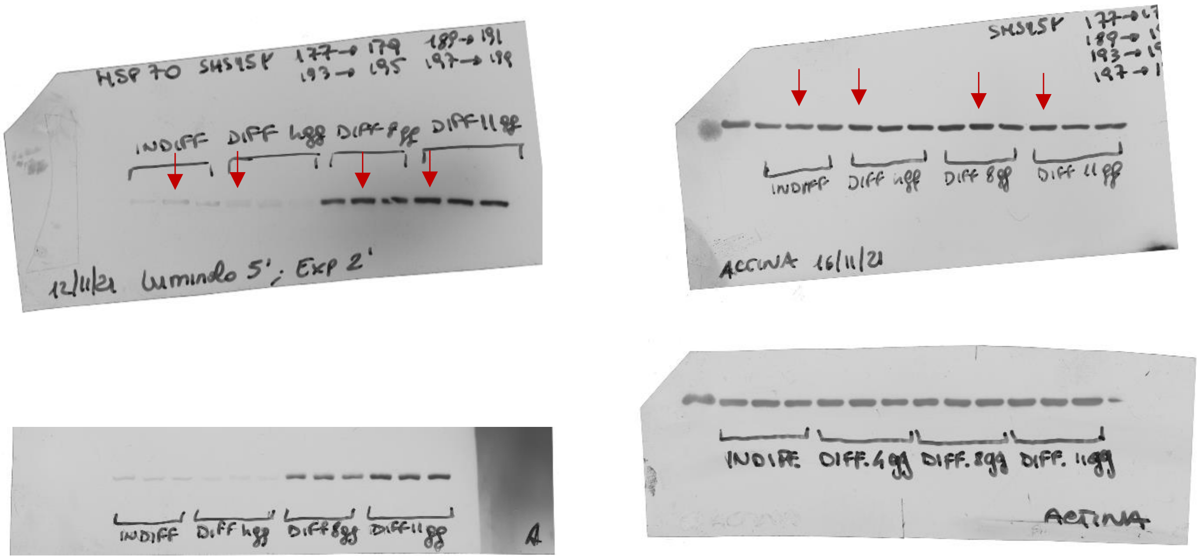

Figure3C-D

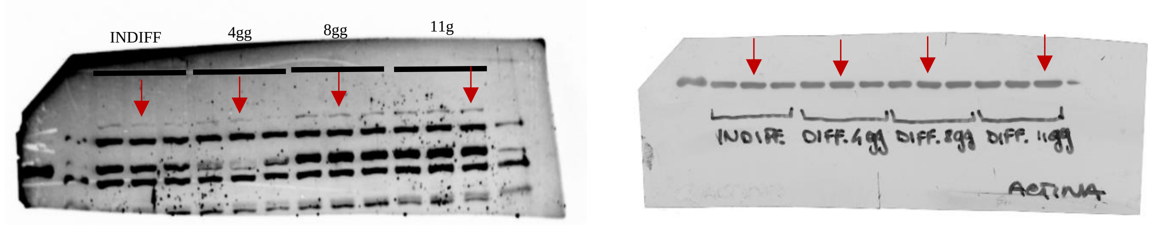

Figure 3E-F

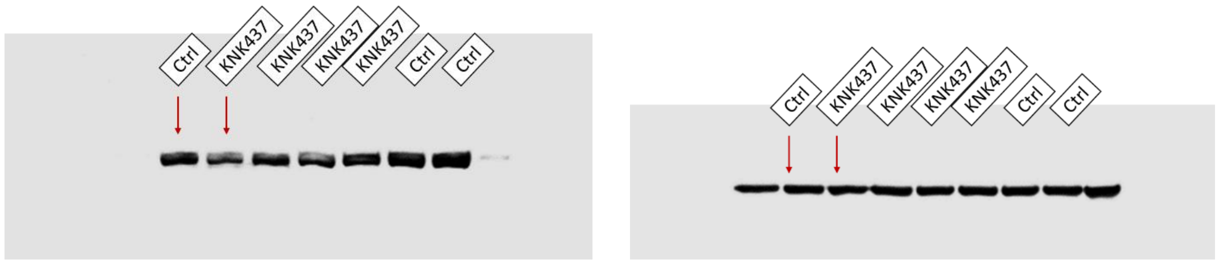

Figure 4D-E

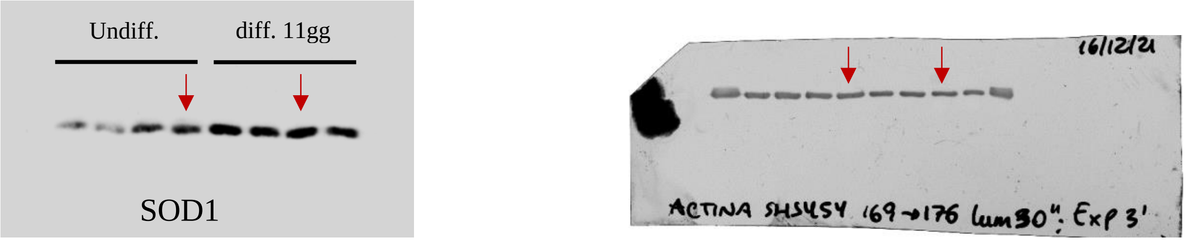

Figure 4F-G

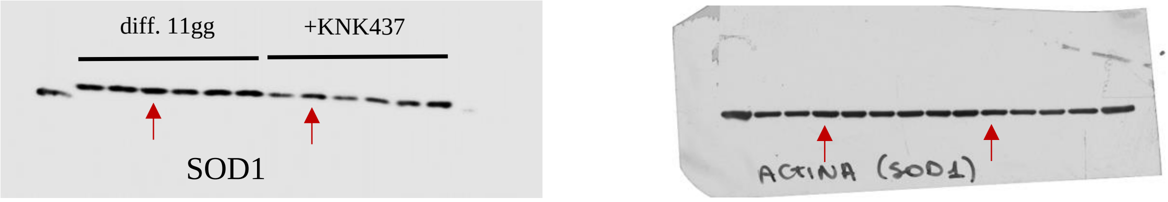

Figure 5A-B

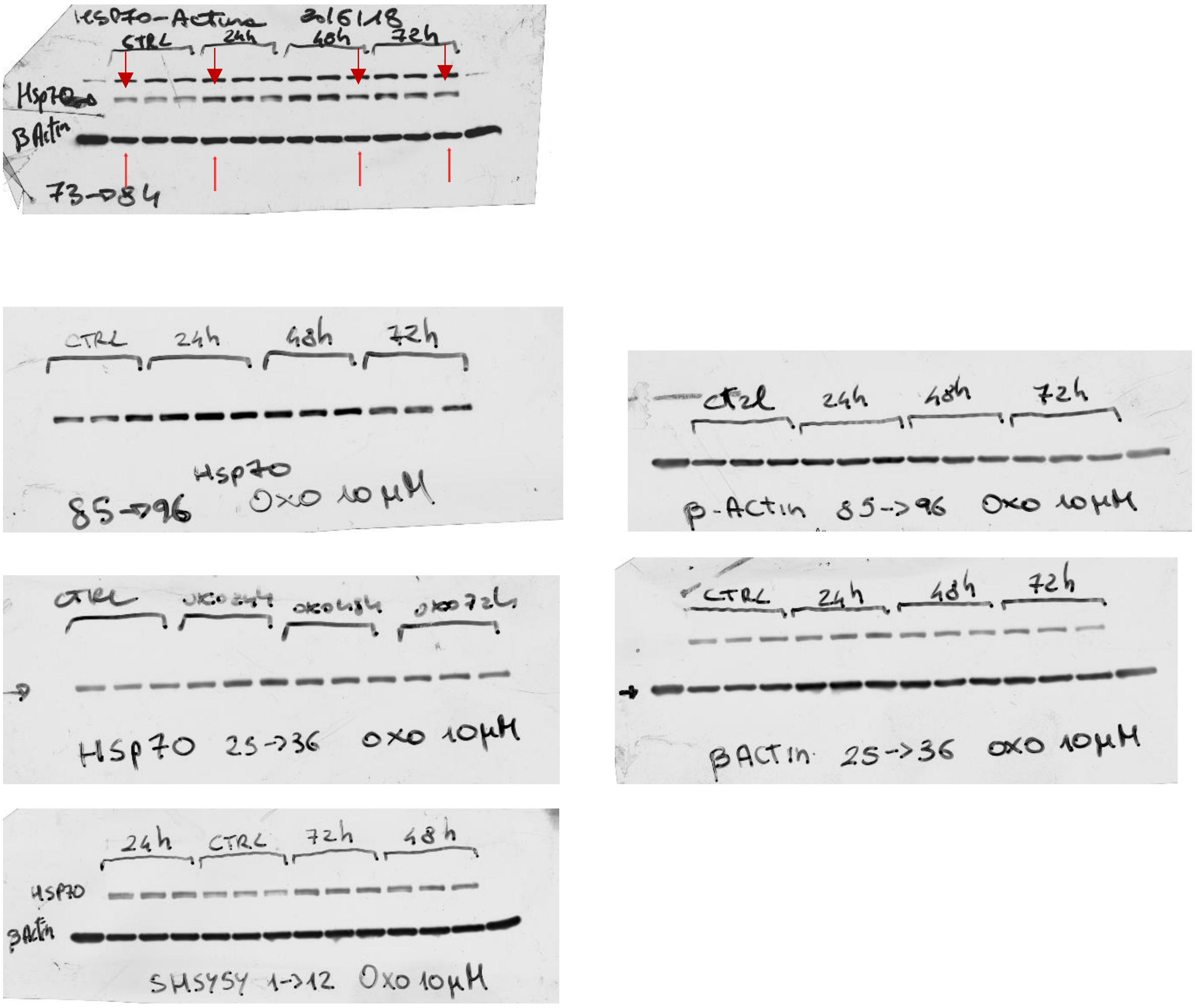

Figure 5C-D

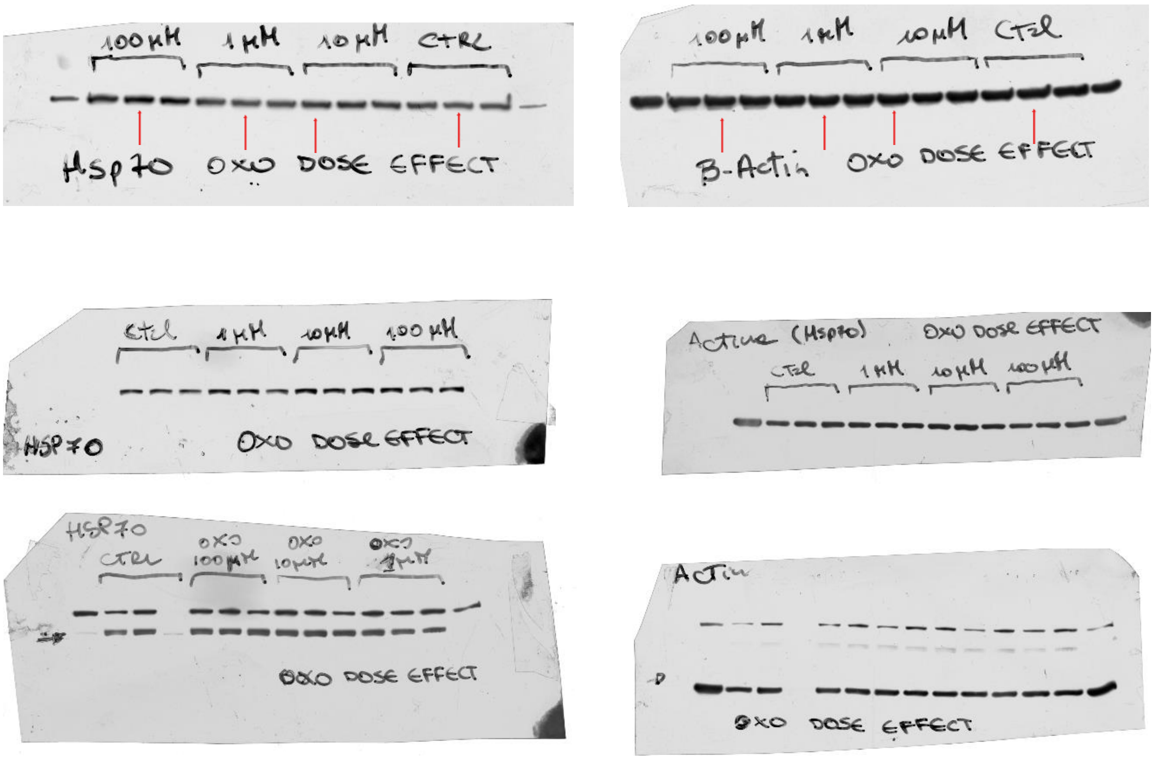

Figure 5G-H

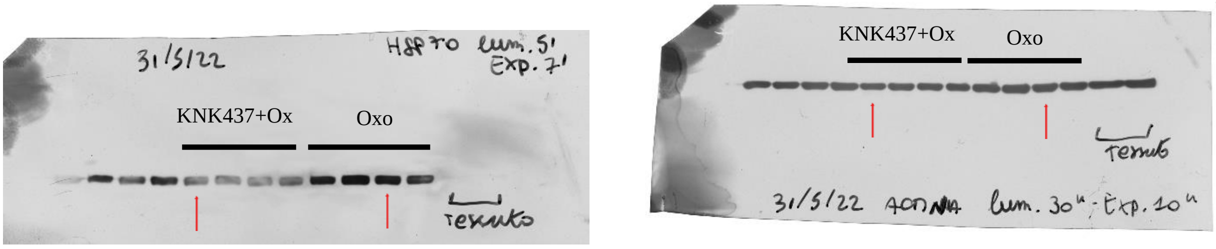

Figure 5E-F

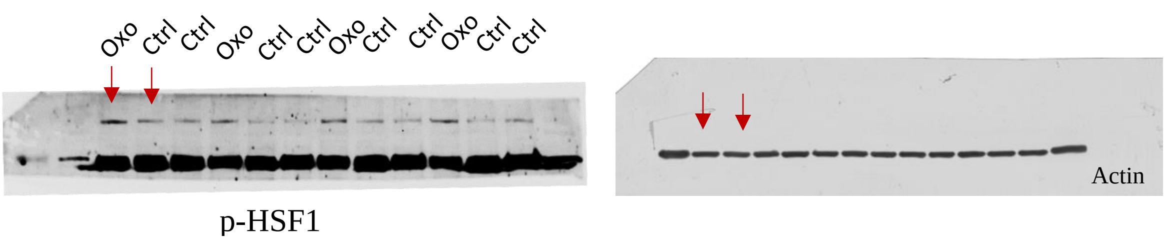

Figure 7A-B

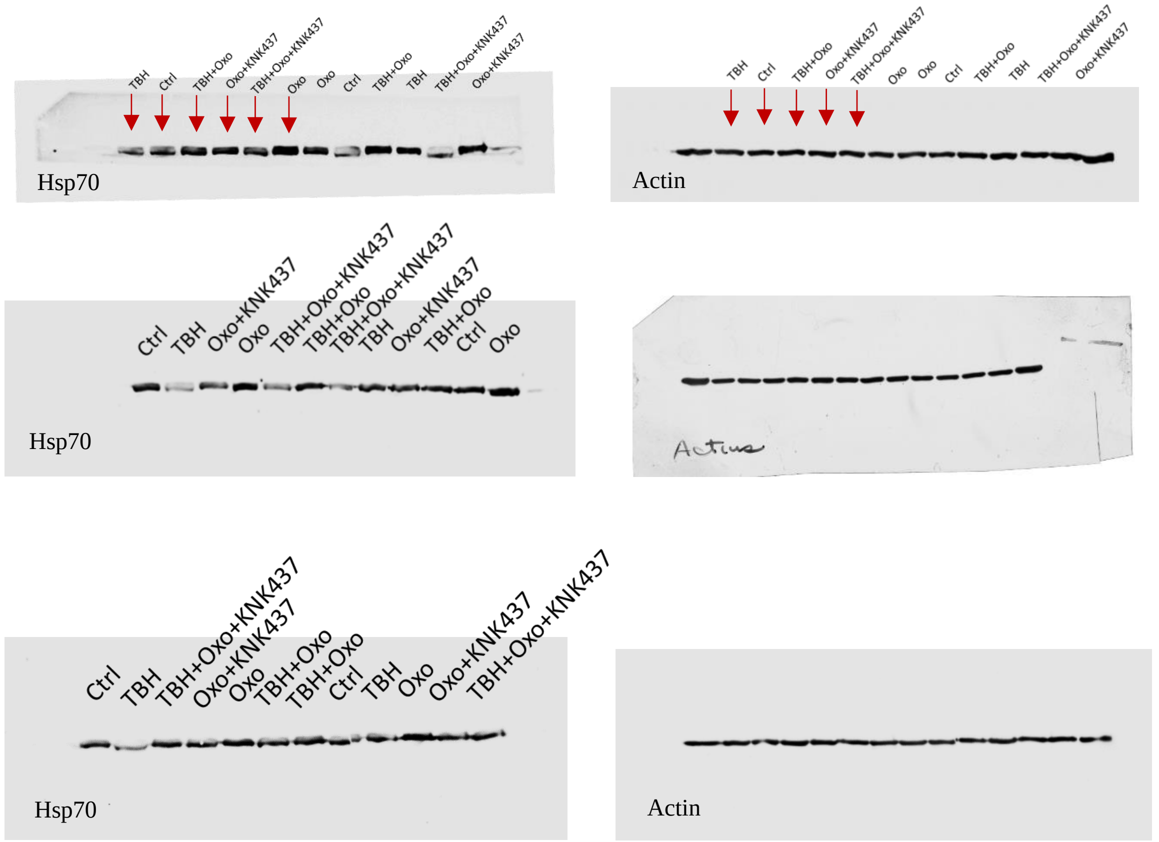

Figure7 C-D

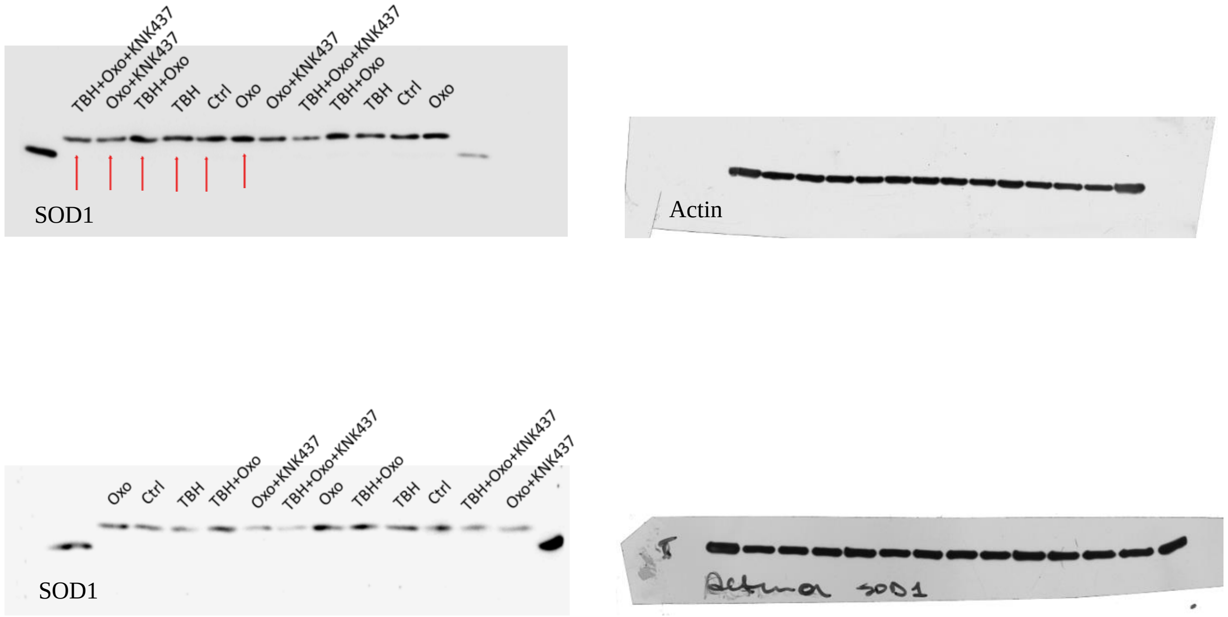

Figure S2

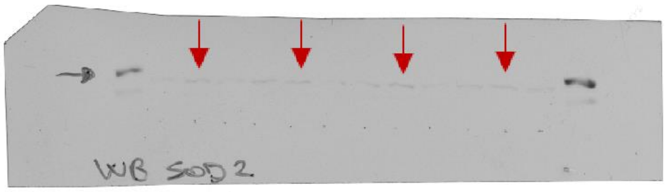

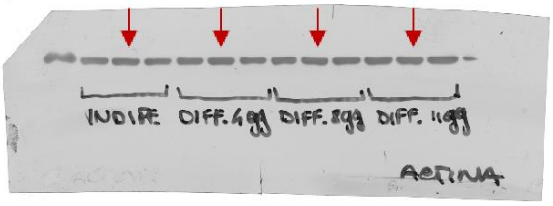

Supplement: Supplementary file 1 [file antioxidants-12-00687-s001.zip › antioxidants-2249833-supplementary.pdf]
